# Taxation Categories for Long-term Care Insurance Premiums and Mortality Among Elderly Japanese: A Cohort Study

**DOI:** 10.2188/jea.JE20120011

**Published:** 2013-01-05

**Authors:** Yoshihisa Fujino, Ryuichi Tanaka, Tatsuhiko Kubo, Shinya Matsuda

**Affiliations:** 1 Department of Preventive Medicine and Community Health, University of Occupational and Environmental Health, Kitakyushu, Fukuoka, Japan; 2 National Graduate Institute for Policy Studies, Tokyo, Japan

**Keywords:** Japan, income, cohort study, aged, long-term care

## Abstract

**Background:**

This cohort study examined the association between taxation categories of long-term care insurance premiums and survival among elderly Japanese.

**Methods:**

A total of 3000 participants aged 60 years or older were randomly recruited in Y City, Japan in 2002, of whom 2964 provided complete information for analysis. Information on income level, mobility status, medical status, and vital status of each participant was collected annually from 2002 to 2006. Follow-up surveys on survival were conducted until August 2007. Hazard ratios (HRs) were estimated by a Cox model, using taxation categories at baseline. In these analyses, age-adjusted and age- and mobility-adjusted models were used.

**Results:**

A significantly higher mortality risk was seen only in the lowest taxation category among men: as compared with men in the second highest taxation category, the HR in the lowest category was 2.53 (95% CI, 1.26–5.08, *P* = 0.009). This significant association between taxation category and mortality was lost after adjustment for mobility. There was no other difference in mortality among taxation categories in men or women.

**Conclusions:**

The present findings only partly supported our hypothesis that taxation category is a good indicator of socioeconomic status in examining health inequalities among elderly Japanese.

## INTRODUCTION

Socioeconomic status is a widely recognized determinant of health, and occupation, education, and income are often used as indices of socioeconomic status.^1^ Generally, those with higher socioeconomic status have better health outcomes, including cardiovascular disease, cancer, and mental health outcomes, regardless of the values of these indices.^2^^–^^9^ Many recent Japanese studies have reported an association between socioeconomic status (often measured by educational level and occupation) and health.^10^^–^^14^

Income is also regarded as a strong determinant of various health outcomes.^1^^,^^15^^,^^16^ For both historical and social reasons, however, obtaining information on income for the purpose of measuring socioeconomic status has been very difficult in Japan. Very few epidemiologic studies conducted in Japan have used income information, and only some studies used self-reported income. While several cross-sectional^17^^–^^21^ and ecologic studies^14^^,^^22^^,^^23^ have reported an association between income and health in Japan, we are unaware of any prospective studies of this association. Examining the impact of income level on mortality among the Japanese population is therefore of considerable interest.

One promising approach is to utilize the taxation category used to determine long-term care insurance premiums as a proxy of income. In general, municipalities use 5 to 10 taxation categories to calculate long-term care insurance premiums, based on an individual’s and his or her family members’ income, including wage and pension income. Information on individual taxation levels based on long-term care insurance premiums can be relatively easily obtained in cooperation with municipal offices.

We argue that taxation category for long-term care insurance premiums is an indirect measure of income and an indicator of socioeconomic status for Japanese elderly adults. There are 2 advantages to the use of taxation category as a proxy of socioeconomic status. First, taxation category is based on the incomes of both the individual and his or her family members. Given that an individual’s affluence depends on both these incomes, a measure that accounts for both may be a better indicator of the individual’s living standards in terms of socioeconomic status. Second, taxation category is widely used in administrative functions by municipal offices throughout Japan and is therefore, in addition to education and occupation, a versatile indicator of socioeconomic status for elderly Japanese.

We prospectively examined the association between individual taxation category for long-term care insurance premiums and survival among elderly Japanese.

## METHODS

### Study participants

The participants were randomly identified from resident registry data of Y city, Fukuoka Prefecture, Japan in 2002. The 3000 identified participants accounted for approximately 10% of residents aged 60 years or older across 5 school districts. Only adults living at home were included; those living in nursing homes were excluded. After exclusion of 27 participants who declined participation, data from 2964 (1241 men and 1723 women) were used in the analysis. The participants were visited at home by trained local welfare commissioners who collected information using a questionnaire administered by face-to-face interview. Annual visits to collect information were conducted from 2002 to 2006, while information about the income and vital status of each participant was provided by the city municipal office. Follow-up surveys of survival were conducted until August 2007. Participants were censored on the date of death or 31 August 2007, whichever came first, for a total of 13 486 person-years of follow-up (5508 person-years for men and 7978 person-years for women). During the 5-year follow-up period, a total of 427 deaths (233 men and 194 women) were recorded.

This study was approved by the Ethics Committee of the University of Occupational and Environmental Health, Japan. All participants provided informed consent to participate.

### Measurement

Five taxation categories for long-term care insurance premiums were used, namely level 1, welfare recipient; level 2, participant and all family members are excluded from taxation; level 3, participant is excluded from taxation, but family members are subject to taxation; level 4, participant is subject to taxation and has an income of about 2 000 000 JPY or less; and level 5, participant is subject to taxation and has an income of more than about 2 000 000 JPY (Figure).

**Figure.**
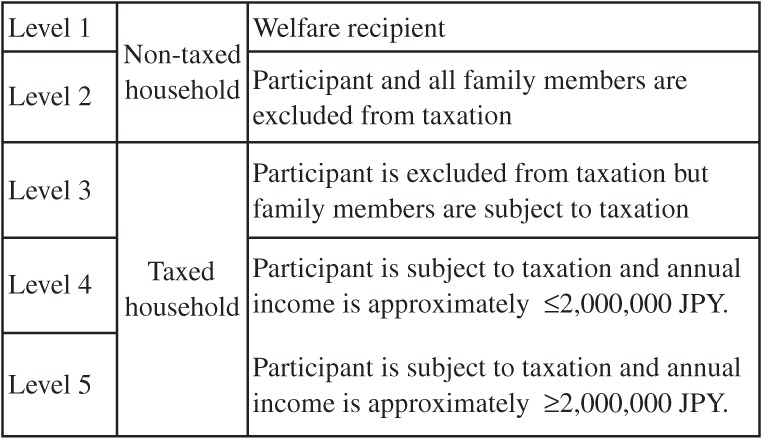
Taxation categories for long-term care insurance premiums for adults aged 65 years and older

Mobility status was measured according to the Typology of the Aged with Illustrations, a validated instrument for the measurement of elderly function,^24^^–^^28^ using the following definitions: level 5, can climb stairs without aid or assistive devices; level 4, cannot climb stairs without aid but can walk on flat surfaces without aid or assistive devices; level 3, cannot walk on a flat surface without aid but can move around using assistive devices and change position independently while seated; level 2, cannot move around while seated using an assistive device or aid from others but can sit up and maintain a seated position; level 1, cannot sit up or maintain a seated position but can roll over without aid on a bed; and level 0, cannot roll over on a bed without aid while lying down.

### Statistical analysis

Hazard ratios (HRs) were estimated by a Cox model, using taxation categories at baseline. These analyses used age-adjusted and age- and mobility-adjusted models. Because data for several possible confounding factors were unavailable, we used mobility as a proxy indicator of general health to adjust for health status at baseline. Data were analyzed using STATA statistical software, version 12 (Stata Corporation, College Station, TX, USA).

## RESULTS

Table 1 shows baseline characteristics according to taxation category and sex. Participants receiving welfare were older than those in the other groups, had a lower level of mobility, and were more likely to be hospitalized. Among men and women, participants in higher taxation categories were younger and healthier than those in lower taxation categories.

**Table 1.** Baseline characteristics of participants according to taxation category for long-term care insurance premiums

	Taxation category, Men (*n* = 1241)					Taxation category, Women (*n* = 1723)				
	
	level 1	level 2	level 3	level 4	level 5	level 1	level 2	level 3	level 4	level 5
No. of subjects	19	311	171	611	129	57	612	947	84	23

Mean age	78.4	74.8	75.3	75.4	74.5	79.6	76.6	76.3	74.6	76.9
SD	1.0	0.1	0.2	0.1	0.2	0.5	0.1	0.1	0.3	0.5

Mobility^a^ (%)										
level 5	11	78	78	88	95	35	77	76	88	78
level 4	11	9	8	7	5	13	13	13	7	13
level 3	16	6	5	3	0	16	4	5	0	4
level 2	0	0	1	0	0	4	0	0	0	4
level 1	5	0	1	0	0	2	0	1	0	0
level 0	58	6	8	2	1	31	6	5	5	0

Medical status (%)										
Inpatient	63	6	8	2	1	31	5	5	5	0
Receiving periodic outpatient treatment	37	62	62	62	57	60	67	63	55	70
Not receiving medical care	0	32	30	36	43	9	28	32	40	30

^a^level 5, can climb stairs without aid or assistive devices; level 4, cannot climb stairs without aid but can walk on a flat surface without aid or assistive devices; level 3, cannot walk on a flat surface without aid but can move around using assistive devices and perform transfer independently while seated; level 2, cannot move around or transfer while seated using assistive devices or with aid from others but can sit up and maintain a seated position; level 1, cannot sit up or maintain a seated position but can roll over on a bed without aid; and level 0, cannot roll over on a bed without aid while lying down.

Age-adjusted HRs of mortality according to baseline taxation category are shown in Table 2. As compared with men in the second highest category, the HR of the lowest taxation category was 2.53 (95% CI, 1.26–5.08, *P* = 0.009) and that of the second lowest taxation category was 1.31 (95% CI, 0.97–1.77, *P* = 0.079). However, when the model included mobility at baseline, taxation category was not significantly associated with mortality in men. In women, neither the age- nor age- and mobility-adjusted model showed any significant association between taxation category and mortality.

**Table 2.** Hazard ratios (HR) for mortality according to baseline taxation category for long-term care insurance premiums

	Men								Women							
	
	Age-adjusted				Multivariate				Age-adjusted				Multivariate			
			
	HR	95% CI		*P*	HR	95% CI		*P*	HR	95% CI		*P*	HR	95% CI		*P*
Taxation category																
level 1	2.53	1.26	5.08	0.009	1.68	0.76	3.68	0.197	1.36	0.51	3.67	0.541	0.71	0.25	1.99	0.510
level 2	1.31	0.97	1.77	0.079	1.16	0.85	1.58	0.344	1.04	0.45	2.41	0.923	1.03	0.44	2.38	0.952
level 3	1.03	0.69	1.52	0.886	0.93	0.62	1.39	0.734	1.10	0.48	2.50	0.827	0.94	0.41	2.16	0.892
level 4	Reference				Reference				Reference				Reference			
level 5	0.88	0.52	1.50	0.647	1.00	0.59	1.69	0.990	1.83	0.52	6.49	0.350	1.83	0.51	6.55	0.355

Mobility^a^																
level 0					3.09	1.82	5.24	<0.001					6.17	3.88	9.81	<0.001
level 1					1.38	0.29	6.47	0.687					4.74	1.64	13.68	0.004
level 2					7.29	2.30	23.03	0.001					4.65	1.60	13.50	0.005
level 3					3.38	2.18	5.25	<0.001					2.37	1.33	4.22	0.004
level 4					2.30	1.58	3.37	<0.001					2.71	1.82	4.05	<0.001
level 5					Reference								Reference			

Age	1.09	1.07	1.11	<0.001	1.07	1.05	1.09	<0.001	1.13	1.11	1.15	<0.001	1.08	1.06	1.10	<0.001

^a^level 5, can climb stairs without aid or assistive devices; level 4, cannot climb stairs without aid but can walk on a flat surface without aid or assistive devices; level 3, cannot walk on a flat surface without aid but can move around using assistive devices and perform transfer independently while seated; level 2, cannot move around or transfer while seated using assistive devices or with aid from others but can sit up and maintain a seated position; level 1, cannot sit up or maintain a seated position but can roll over on a bed without aid; and level 0, cannot roll over on a bed without aid while lying down.

We also obtained information on income in 2006. Table 3 shows the amount of income according to taxation category. For both men and women, the 10th income percentiles of the level 4 and 5 categories were about 1 300 000 JPY and 2 000 000 JPY, respectively. In addition, all participants in the level 4 and 5 categories received more than 600 000 JPY in non-pension income. These results indicate that men and women at levels 4 and 5 were economically active and may have had regular work. Income distributions between the 10th and 90th percentiles largely overlapped for levels 2 and 3, and for levels 4 and 5, in men and women.

**Table 3.** Income according to taxation category for long-term care insurance premiums at the 5th wave

Taxation category^a^	Total income				Non-pension income		
	
	Mean (JPY)	10th percentile (JPY)	90th percentile (JPY)	Non-pension >600 000 JPY (%)	Mean (JPY)	10th percentile (JPY)	90th percentile (JPY)
Men							
level 1	—	—	—	—	—	—	—
level 2	2 371 134	700 332	3 756 392	52	594 067	0	1 178 996
level 3	2 321 973	627 496	3 708 456	52	576 949	0	1 170 300
level 4	4 530 586	3 965 252	5 303 243	100	1 570 847	1 296 036	1 880 500
level 5	6 589 896	4 981 066	9 083 326	100	3 197 355	2 074 758	4 912 153

Women							
level 1	—	—	—	—	—	—	—
level 2	717 261	0	1 289 796	4	66 429	0	178 243
level 3	598 267	0	1 130 932	2	43 059	0	81 000
level 4	4 069 679	2 149 164	4 924 721	100	1 530 114	1 332 772	1 834 496
level 5	6 648 308	2 916 742	11 500 000	100	5 202 583	2 185 339	9 439 120

^a^Taxation categories: level 1, welfare recipient; level 2, participant and all family members excluded from taxation; level 3, participant is excluded from taxation but family members are subject to taxation; level 4, participant is subject to taxation and income is about 2 000 000 JPY or less; and level 5, participant is subject to taxation and income is more than about 2 000 000 JPY.

## DISCUSSION

In this study, mortality risk was higher only among men in the lowest taxation category. There was no such association among women. We believe that the association among men cannot be attributed to income only because the lowest taxation category consisted of men receiving welfare due to unemployment, economic distress, and/or health problems and disabilities. This is supported by the fact that the association was weakened by adjustment for mobility, which is considered a proxy of general health, and by the fact that mobility itself was significantly associated with mortality.

Except for the lowest category, there was no mortality difference among taxation categories. If taxation category truly reflects individual socioeconomic status, then the present study indicates that there is no obvious disparity in mortality among different socioeconomic groups in Japan. This implies that a change in classification from groups based on crude individual income to groups based on taxation for long-term care insurance premium may mitigate putatively inherent health disparities between different income groups. This mitigation probably acts via the impact of various social benefits such as the social security system and taxation policy, ie, a lower taxation category may receive more social benefits than a higher taxation category.

There are several possible reasons for the lack of association in this study. First, taxation categories for long-term care insurance premiums do not necessarily parallel individual income, as shown in Table 3. Taxation categories are based on not only an individual’s income but also on that of family members, and some components of overall income are excluded from taxation in the complex formulas used in the calculations. In particular, the income distributions of level 2 and 3 participants largely overlapped.

Second, individual income is not a sensitive indicator of the economic status of retirees. A study suggested that the relationship between income and health weakens after age 65.^29^ In addition, Allin et al suggested that, in determining socioeconomic status of older adult populations that include both economically active and inactive individuals, wealth is a better measure than income.^30^ We did not measure participants’ assets and liabilities, so individual wealth could not be measured.

Third, we did not distinguish between work income and other income. Different sources of income may have varying impacts on health. As shown in Table 3, women at levels 4 and 5 were regarded as currently working, while those at level 3 and lower were not. However, levels 4 and 5 accounted for only 6% of women. In contrast, about half of men at levels 2 and 3 had more than 600 000 JPY in non-pension income. We expect that most people who received 600 000 JPY annually in non-pension income, the equivalent of 50 000 JPY per month, were currently working. The taxation category for long-term care insurance premiums is not a good indicator of the economic activity of people.

Further limitations of our study warrant mention. We did not obtain information on a number of important possible confounding and pathway variables, including smoking, drinking, physical exercise, nutritional intake, stress, medical diagnoses, educational status, or formal care, including institutionalized care. This lack of adjustment weakens any conclusions concerning causal relationships, which are in any case beyond the scope of this study. In addition, the relatively short observation period may have resulted in latent bias in the results.

In conclusion, we found that male welfare recipients had a higher mortality risk, which was mainly due to mobility status. Except for the lowest taxation category of men, there was no difference in mortality among taxation categories among men or women. These results thus do not support our hypothesis that taxation category is a good indicator of socioeconomic status in examining health inequalities among elderly Japanese. Further study is needed to determine whether taxation category is a sufficiently sensitive socioeconomic indicator of health inequalities among elderly Japanese, using various health measurements. In addition, in relation to individual income, use of taxation categories for long-term care insurance premiums may not be a valid measurement. Evaluation of health disparities by income in Japan requires further study using accurate measurement of individual income.
